# Response to: Comment on “Assessing Effectiveness and Costs in Robot-Mediated Lower Limbs Rehabilitation: A Meta-Analysis and State of the Art”

**DOI:** 10.1155/2019/9693801

**Published:** 2019-09-24

**Authors:** Giorgio Carpino, Alessandra Pezzola, Michele Urbano, Eugenio Guglielmelli

**Affiliations:** ^1^Laboratory of Biomedical Robotics and Biomicrosystems, Università Campus Bio-Medico di Roma, I-00128 Rome, Italy; ^2^Polyclinic General Direction, Università Campus Bio-Medico di Roma, I-00128 Rome, Italy

The authors have analysed the comments by Alberto Esquenazi [1] on their article “Assessing Effectiveness and Costs in Robot-Mediated Lower Limbs Rehabilitation: A Meta-Analysis and State of the Art” [2]. The authors believe that the investigated topic, and assessment studies on medical devices generally, is a debated theme since the scientific evidence is sparse and the attempts to collect information are challenging. The need of the assessment for medical device is, however, crucial for supporting the decision-making process. The literature in the field of robotics introduced in the clinical procedures to help clinicians to perform their tasks is missing these types of assessment studies.

The replies to the comments on the article are given below.

Regarding the purchasing costs, the comments are agreeable, but it is important not to miss the aims of the study. Starting from a meta-analysis of robot-mediated lower limbs rehabilitation for stroke-affected patients, the core of the article is to evaluate the effectiveness of the robotic approach through the use of wearable robots or operational machines with respect to the conventional through a series of outcomes (including a cost-effectiveness analysis) with the final objective to set up some general guidelines for the decision maker for the choice of best procedure to follow for the motor rehabilitation. It is, thus, not appropriate to lessen the scope of the article to a comparison between the prices of the mentioned medical class of devices. In addition, the study is based on references and costs up to 2015 (the study was set up at the end of 2015; then, for delays in the finalization of the paper and in the review procedures of previously selected journals, it has been published in 2018). The costs of the devices were modified in these years, and the authors are happy to observe that there is a levelling of the device cost. This is the natural lifecycle of a medical device and, in general, of a product. In this new price configuration, comparing state-of-the-art wearable robots and state-of-the-art operational machines, the choice of the decision makers becomes more difficult, and this is the natural business competition between companies and, thus, the authors are not convinced that modifying the calculations in the article can bring to an added value with respect to its scope. In a year from now, for example, considering new data and new device coming from new studies, the costs of the device and probably the outcomes of the study itself will change again, while the methodology of the study will be the same in time.

On the number of human resources, they are calculated as the average from the data reported in the references included in the study. Regarding the other two articles cited by Esquenazi [3, 4], it is not clear if they fall in the same inclusion criteria described in Section 2.1 of the article [2]. If only the intensive training is considered, the number of necessary human resources goes up. This means that the difference between the costs calculated in the robotic therapy and in the conventional therapy decreases and, thus, the conclusion that the robotic therapy is more cost efficient with respect to the conventional therapy is even more verified. Furthermore, the study of Esquenazi et al. [4] is not reported in the article since it is posterior to the references analysed in the study, as already described above. In this case, where the number of human resources adopted for the operational machine-based therapy is higher than the one in the wearable robots therapy, it depends if [4] fits in the same inclusion criteria or not, as already stated.

Regarding the cost of the treadmill, for the conventional therapy the treadmill cost was not considered because the authors assumed that this device is generally already present in a rehabilitation structure and also because its cost was considered less than 1.000 €. Splitting this cost for the number of sessions, a marginal cost comes up.

Concerning the comment on the possibility for the robotic therapy to allow parallel sessions, thus increasing the amount of therapy sessions that a single therapist can provide, the authors agree on this comment; however, it much depends on the work management of the individual rehabilitation clinics. It seems that this is more of a suggestion to give to the decision maker than a hypothesis to put in the methodological flow of the study.

On the safety issues, the sentence is based on the evidence in Cenciarini and Dollar [5], but it also considers the study of Jarrassé et al. [6] on the upper limbs that was already cited in other studies by the authors, i.e., on the design of a nonanthropomorphic wearable robots for lower limbs [7]. The conclusion starts from the analysis of the outcome on the timing of the rehabilitation session and, as described in the article, in the case of wearable robots-mediated therapy, a considerable time is spent to set up the robot. This applies even more in the exoskeletons case, in general, since the risk to not properly set the device and create some misalignments between the robotic and human joints is high. Such misalignments can cause the exchange of unwanted interaction forces causing discomfort or even pain for the patient. This situation could be dangerous to the patient, as well as an obstacle to his/her movement; however, this is a rare occurrence in the case of operational machines. The countermeasures, hardware and software, introduced in the Lokomat system are very important to mitigate the abovementioned possible risks. With respect to operational machines and patients with severe impairments, the authors agree with the comment by Esquenazi, although this case seems to not meet the inclusion criteria of the study.

Regarding the update of the Cochrane review, the authors are aware of this but, as already stated, the study was drawn before 2017. Regarding the comment on 26 or 28 studies considered in the article, the motivation is that 2 of them (Kelley et al. [8] and van Nunen et al. [9]) are an update of two studies already considered in the 2013 version of the Cochrane. In this view, it seems that only one study (Calabrò et al. [10]) is not considered in the version 2017 of the Cochrane, and it is difficult for the authors to reply to this comment. We appreciate this doubt being raised and the opportunity to better explain this methodological aspect.

The authors hope to have replied in a clear way to the comments by Esquenazi highlighting the key points of the methodology applied to the study. Of course, things change over time and will change fast, but the authors hope to have setup guidelines for the decision makers in order to give them the tools to choose the best technology for the medical procedures at the time being.
